# A method for discovery of transcription factors controlling *Brucella* sRNAs

**DOI:** 10.1128/spectrum.01909-25

**Published:** 2025-11-13

**Authors:** Mitchell T. Caudill, Jillian R. Marshall, Clayton C. Caswell

**Affiliations:** 1 Center for One Health Research, Department of Biomedical Sciences and Pathobiology, VA-MD College of Veterinary Medicine, Virginia Tech 229659, Blacksburg, Virginia, USA; Forschungszentrum Jülich GmbH, Juelich, Germany

**Keywords:** *Brucella*, sRNAs, transcriptional regulation

## Abstract

In the genus *Brucella*, approximately 40 small regulatory RNAs (sRNAs) have been identified and validated via northern blot analysis, but the transcription factors controlling sRNA transcript levels are largely unknown. Here, we present a method for more rapidly discovering conditions and transcription factors controlling sRNA levels from transcriptomic data sets. Using this approach, we reanalyzed publicly available RNA-seq data sets and generated a large list of predicted regulatory relationships. Northern blot analyses confirmed several of these predictions, covering a wide range of potential transcription factors. Specific findings include that VjbR, a major quorum-sensing regulator of virulence in *Brucella*, controls levels of the MavR sRNA, and the alternative sigma factor RpoE1 is absolutely required for the expression of *bsr6* while also contributing to Bsr8 production during oxidative stress. We also found that RNase E is linked to transcript levels of Bsr7, and the transcriptional regulator PhyK modulates the expression of two *Brucella* sRNAs. Overall, the methods described here can be used in future transcriptomic studies to predict and validate transcriptional regulators and conditions that influence sRNA levels in bacteria.

**IMPORTANCE:**

Regulatory small RNAs control translation for numerous proteins linked to virulence in pathogenic bacteria, but have proven difficult to fully characterize. We developed a new method to discover transcription factors and conditions controlling small regulatory RNAs (sRNA) expression. This method can be applied to other bacteria and future *Brucella* transcriptomic studies to aid in understanding pathogenesis and virulence factors mediated by sRNAs.

## INTRODUCTION

Bacteria adapt to shifting environmental constraints by altering genetic transcription to survive or replicate in the new conditions. Typically, transcription and translation are coupled in bacteria, allowing simultaneous generation of mRNA transcripts and proteins (1). Small regulatory RNAs (sRNAs) allow for decoupling of this process by providing an additional regulatory layer. The sRNA–mRNA interaction allows the bacterium to conserve critical resources while still maintaining the ability to rapidly respond to changing environments and fitness landscapes (2, 3).

The regulatory actions of sRNAs are involved in the expression of genes controlling survival and virulence in many pathogenic bacteria (2, 4). Despite the importance of this regulation to pathogenic bacteria, the discovery and characterization of sRNAs has lagged far behind the characterization of bacterial proteins. One potential reason for this is the lack of an obvious start and stop sequence within the DNA, necessitating the discovery of the sRNA through whole transcriptomic mapping. After discovery, validation can be laborious with verification of the transcript via northern blotting, and then determination of the exact sequence via rapid amplification of cDNA ends or a similar technique (5, 6). Even once discovered and validated, sRNAs are rarely annotated within reference genomes, limiting the information gathered about them from transcriptomic studies (7).

Our laboratory studies the genus *Brucella*, whose classical bacterial species are causative agents of brucellosis, a chronic zoonotic disease with significant morbidity (8, 9). Within bacteria of the genus *Brucella,* multiple sRNAs have been identified, with a total of approximately 40 sRNAs having been validated via northern blot analysis. However, only a handful of these sRNAs have been characterized as to their functional role or the regulatory mechanisms controlling their expression (2, 10, 11).

In order to discover transcription factors influencing known and validated sRNA transcript levels, we developed an approach to speed the reanalysis of publicly available RNA-seq data sets. We mapped the transcripts from these data sets to a custom reference sequence that consisted of concatenated DNA sequences from regions containing known and validated *Brucella* sRNAs. By qualitatively comparing the transcript levels of sRNAs from these data sets, we predicted regulatory interactions. For six of these predictions, northern blot analysis confirmed five of the relationships and demonstrated mixed results for one. This method may be useful for both future studies of *Brucella* and for other bacteria to predict and validate factors influencing sRNA levels from publicly available transcriptomic data.

## RESULTS

We generated a custom “genome” by concatenating the approximate genetic loci of known *Brucella* sRNAs and mapping *Brucella* transcriptomes to this file. The sRNA maps and list of transcriptomes examined are available in supplemental files. By overlaying the maps of transcriptomes and associated conditions, we were able to infer regulatory relationships of known small RNA (2A is provided as an example). We used a subjective cut-off of greater than 4× change in normalized mapped reads to predict a regulatory relationship.

**Fig 1:**
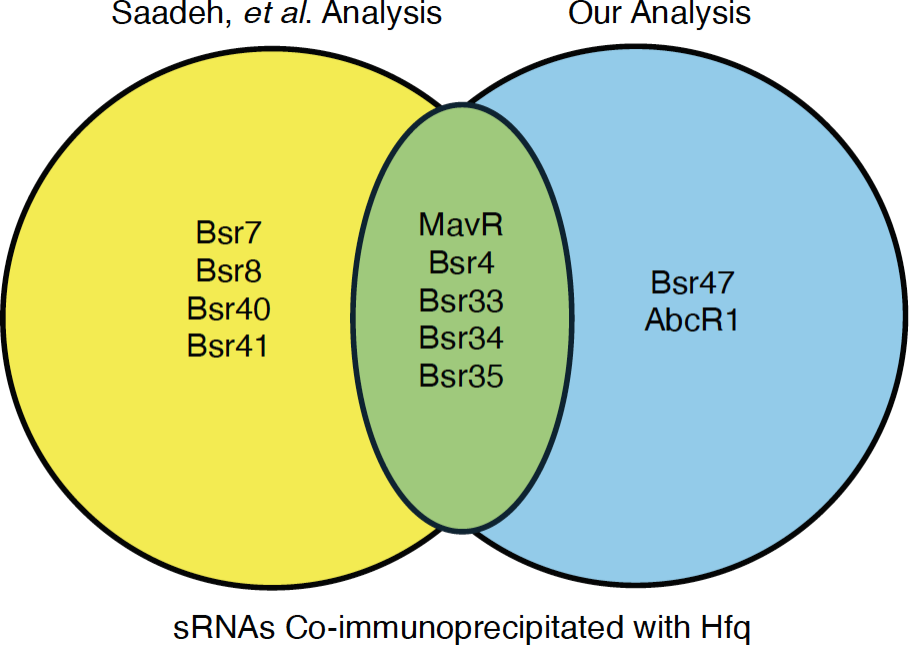
Validation of transcriptome mapping approach. We compared the results of our analysis of the RNA-sequencing data generated by Saadeh et al. to the sRNAs identified in the original analysis. Those in the yellow circle on the left were documented just by Saadeh et al., while those in the blue circle on the right were identified just via our analysis. Those in the overlapping green area were identified by both analyses.

To compare this approach to other pipelines, we compared the results of our analysis pipeline to those of Saadeh et al. (12), which identified sRNAs co-immunoprecipitated with the RNA chaperone Hfq (Fig. 1). In general, we found a good overlap between the analyses. Of the nine sRNAs validated via northern blot in Saadeh et al., five came out of our re-examination of the sequencing data, and of the four remaining sRNAs, three showed association with Hfq in our analysis but were below our 4×-fold change cut-off. We additionally identified two new sRNAs not identified in Saadeh et al. (i.e., AbcR1 and Bsr47) that were independently found to be Hfq-associated (13, 14).

We reexamined numerous RNA-seq data sets with our method to find potentially differentially expressed sRNAs (see supplement for full list). The predicted regulatory relationships between sRNAs and experimental conditions for all transcriptomes examined are shown in Table 1. Bolded predictions are those with associated northern blots in this analysis. The associated northern blots cover multiple types of transcription factors, including a quorum-sensing transcriptional regulator (VjbR), an alternative sigma factor (RpoE1), a histidine kinase (PhyK), and an RNase (RNase E).

**TABLE 1:** Predicted regulatory relationships for *Brucella* sRNAs^*a*^

Condition	sRNA	FC	Species	Source	Condition	sRNA	FC	Species	Source
∆*baaR*	Bsr6	−4	*abortus*	(15)	∆*irr*	Bsr46	−3.5	*melitensis*	(16)
∆*lovHK*	Bsr6	−2.5	*abortus*	(17)	∆*otpR*	Bsr8	2.5	*melitensis*	(18)
∆*lovHK*	Bsr7	−2.5	*abortus*		∆*otpR*	Bsr1	2.5	*melitensis*	
∆*rpoE1*	Bsr53	−2	*abortus*		∆*otpR*	Bsr50	2	*melitensis*	
∆*rpoE1*	Bsr8	−2.5	*abortus*		∆*essRS*	Bsr7	−2.5	*ovis*	(19)
**∆** ***rpoE1***	**Bsr6**	**−3**	*abortus*		∆*phyK*	Bsr48	3.5	*ovis*	(20)
**∆** ***vjbR***	**MavR**	**−2**	*abortus*	(21)	**∆** ***phyK***	**Bsr4**	**3**	*ovis*	
∆*rnaseJ*	Bsr10	3	*abortus*		∆*phyK*	Bsr40	2.5	*ovis*	
*rne*-tnc	Bsr4	4*	*abortus*	(22)	∆*phyK*	Bsr50	2.5	*ovis*	
***rne*** **-tnc**	**Bsr7**	**2.5**	*abortus*		∆*phyK*	Bsr15	2.5	*ovis*	
Mϕ	Bsr48	7	*abortus*	(23)	∆*phyK*	Bsr12	2	*ovis*	
Mϕ	Bsr45	4	*abortus*		∆*phyK*	MavR	2	*ovis*	
Mϕ	Bsr35	3	*abortus*	** **	**∆** ***phyK***	**AbcR2**	**−3.5**	*ovis*	
Mϕ	Bsr46	3	*abortus*	** **	SDS	Bsr48	4	*ovis*	
Mϕ	Bsr3	2	*abortus*		SDS	Bsr40	3.5	*ovis*	
Mϕ	Bsr39	−2	*abortus*		SDS	Bsr35	3	*ovis*	
Mϕ	Bsr33	−2	*abortus*		SDS	Bsr51	3	*ovis*	
Mϕ	Bsr41	−2	*abortus*		SDS	Bsr21	3	*ovis*	
Mϕ	MavR	−2	*abortus*		SDS	Bsr4	3	*ovis*	
Mϕ	Bsr22	−2	*abortus*		SDS	MavR	3	*ovis*	
Mϕ	Bsr1	−2.5	*abortus*		SDS	Bsr35	2.5	*ovis*	
Mϕ	Bsr10	−3	*abortus*		SDS	Bsr46	2.5	*ovis*	
Mϕ	Bsr12	−3	*abortus*		SDS	Bsr19	2.5	*ovis*	
Mϕ	Bsr6	−3	*abortus*		SDS	Bsr7	2.5	*ovis*	
Mϕ	Bsr120	−4	*abortus*		SDS	Bsr36	2	*ovis*	
Mϕ	Bsr4	−4	*abortus*		SDS	Bsr1	−2	*ovis*	
Mϕ	Bsr8	−4.5	*abortus*		SDS	AbcR2	−3	*ovis*	
∆*clpP*	Bsr45	−2	*abortus*	(24)	5% CO_2_	Bsr4	−2	*ovis*	(25)
∆*clpP*	Bsr46	−3	*abortus*		5% CO_2_	Bsr33	−2.5	*ovis*	
∆*clpP*	Bsr30	−4	*abortus*		∆*mucR*	MavR	−2	*canis*	(26)
∆*clpP*	Bsr6	−2.5	*abortus*		∆*mucR*	Bsr21	−2	*canis*	
∆*clpP*	AbcR2	−2.5	*abortus*		∆*mucR*	Bsr44	−2.5	*canis*	
∆*clpP*	Bsr33	−5	*abortus*		∆*mucR*	Bsr4	−3.5	*canis*	
∆*clpP*	Bsr35	−2.5	*abortus*		∆*mucR*	Bsr47	−2.5	*canis*	
∆*clpP*	Bsr9	−2.5	*abortus*		∆*mucR*	Bsr9	−6	*canis*	
∆*clpP*	BsrH	−2.5	*abortus*		∆*mucR*	Bsr1	−3	*canis*	
∆*clpP*	Bsr23	2	*abortus*		∆*mucR*	Bsr12	−3	*canis*	
∆*clpP*	Bsr24	−3	*abortus*		∆*mucR*	Bsr14	−2.5	*canis*	
					∆*mucR*	Bsr15	−3	*canis*	
					∆*mucR*	Bsr22	−4.5	*canis*	

^*a*^ This table contains the predicted regulatory relationships for northern blot-validated *Brucella* sRNAs. Those with bold text have associated a northern blot in this paper. Presented FC is the Log_2_ fold change of the sRNA levels in the specified mutant or condition relative to the wild-type or untreated condition. “Bsr” series of sRNAs can be examined for synonymous sRNA names in the *Brucella* sRNA Rosetta Stone 2.0 (maintained on our laboratory website: https://caswelllab.com/). *Indicates that the relationship was tested in the original manuscript and the finding was consistent with the prediction. The conditions presented in the table are shorthand approximations, and readers are directed to the methods of the original papers for further details.

Ultimately, of the five predictions for which northern blot analysis was performed in this analysis, four had northern blots in agreement with the transcriptome prediction.

**Fig 2:**
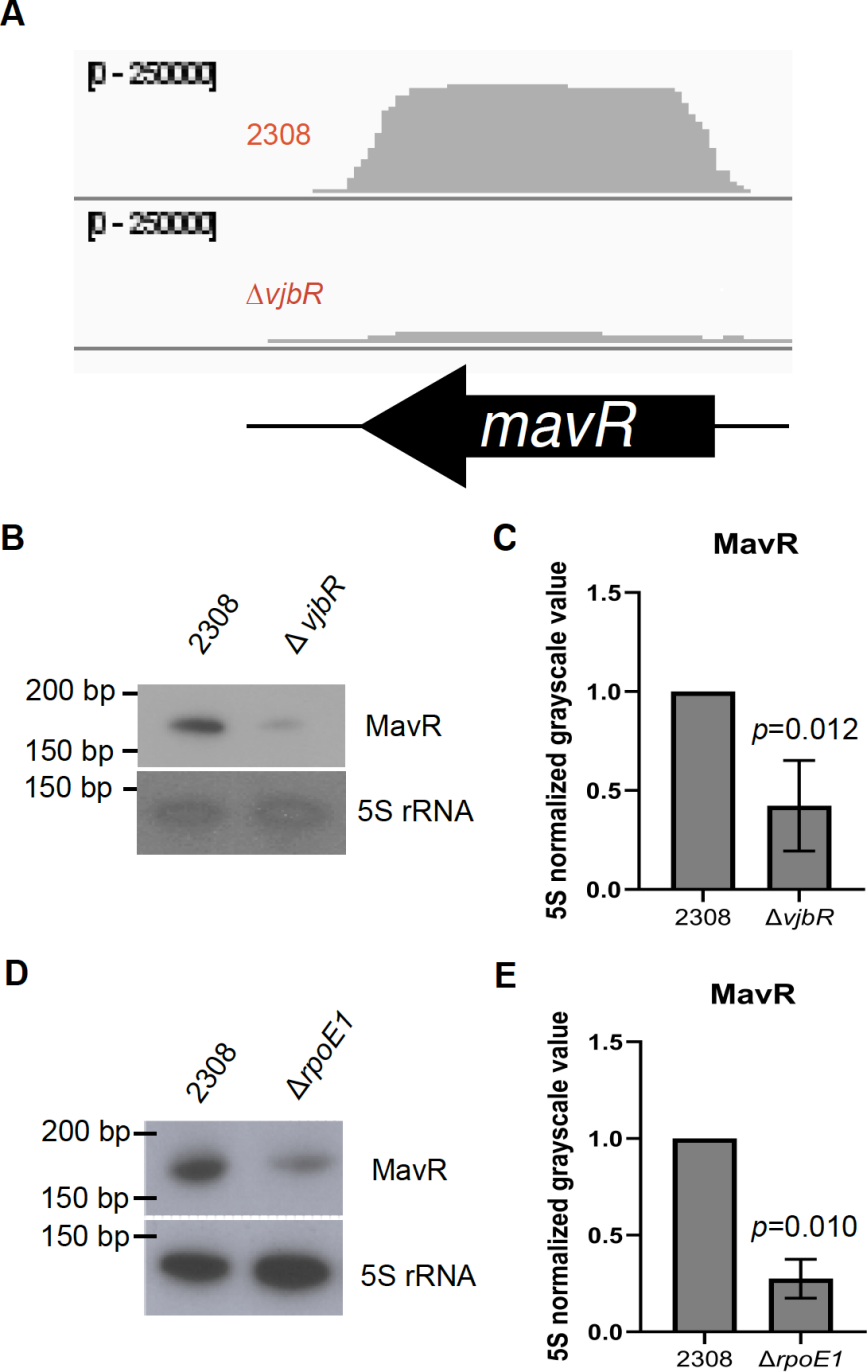
MavR levels are controlled by VjbR and RpoE1. Northern blot analyses and their respective densitometry of 5S rRNA normalized mean grayscale value are displayed. As a representation, panel (**A**) shows the mapped reads of the sRNA MavR as displayed visually in the Integrated Genome Viewer for both wild-type *B. abortus* 2308 and Δ*vjbR* transcriptomes. The scale indicates a maximum of 250,000 reads. Panels (**B and C**) show the relationship between Δ*vjbR* and MavR transcript levels. Panels (**D and E**) show the relationship between Δ*rpoE1* and MavR transcript levels. Each panel is a representative image of northern blot analyses from at least three independent RNA isolations. Statistical analysis was carried out using a two-tailed *t*-test with two-sample equal variance, and the *P* value for each analysis is denoted in the bar graph.

Here and throughout this work, we exclusively utilized *Brucella abortus* 2308 for all of our analyses. This is important to underscore, as the bioinformatic analysis outlined in Table 1 utilized transcriptomic data from a variety of *Brucella* species, and species-related differences need to be considered, which we comment on in later sections of this report. Regarding MavR, we found that the transcript levels of MavR are controlled by VjbR (Fig. 2A through C), with VjbR promoting MavR expression, in line with our prediction. Given that previous work has shown that VjbR is within the RpoE1 regulon (27), we were curious if the MavR transcript was lowered in the Δ*rpoE1* background, as well as the Δ*vjbR* background. We performed this additional northern blot (2D-E), and this analysis revealed that MavR levels are reduced in a *B. abortus* Δ*rpoE1* strain, indicating promotion of MavR levels by RpoE1.

**Fig 3:**
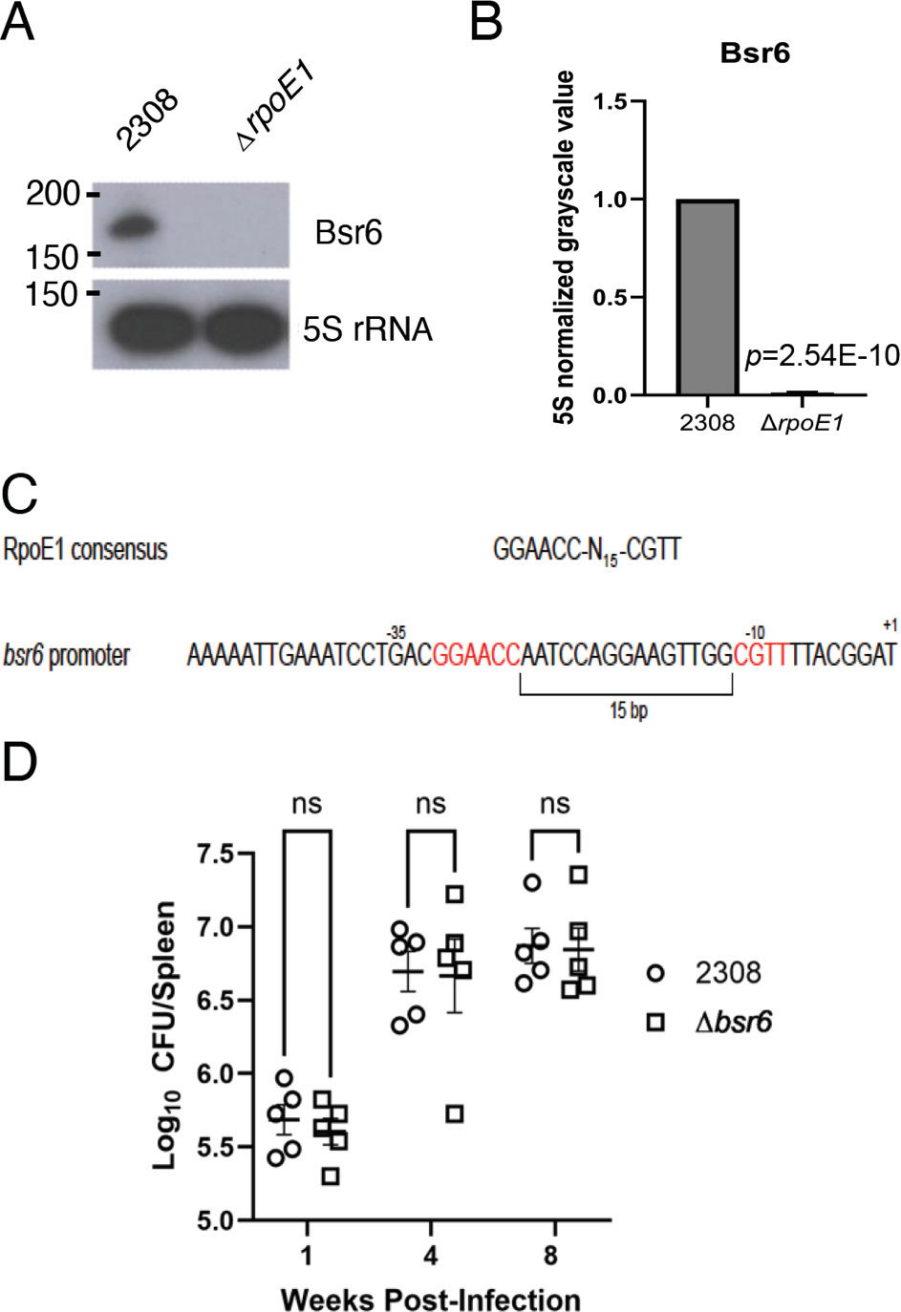
Bsr6 is controlled by RpoE1 but not required for *B. abortus* virulence. Panel (**A**) shows a representative northern blot analysis of Bsr6 transcript levels in the wild-type strain *B. abortus* 2308 and the Δ*rpoE1* strain, and panel (**B**) depicts the densitometry of 5S rRNA normalized mean grayscale values from northern blot analyses carried out from at least three independent RNA isolations. Statistical analysis was carried out using a two-tailed *t*-test with two-sample equal variance, and the *P* value for each analysis is denoted in the bar graph. Panel (**C**) shows the RpoE1 consensus binding site within the *bsr6* promoter. Panel (**D**) displays splenic colony-forming units at the indicated time points for five female BALB/c mice per strain infected via the intraperitoneal route. Statistical testing consisted of a two-way analysis of variance with post hoc Tukey’s multiple comparison. NS indicates no statistical significance.

We also found that RpoE1 controls production of Bsr6 (Fig. 3A and B). Given the clear presence of an RpoE1 binding box in the promoter region of *bsr6* (Fig. 3C), we sought to test whether Bsr6 contributes to the attenuation of the Δ*rpoE1* strain in a mouse model (28, 29). For this, we constructed a *B. abortus* Δ*bsr6* deletion strain and assessed the ability of the mutant strain to infect and colonize BALB/c mice (Fig. 3C). At 1-, 4-, and 8-weeks post-infection, we observed no differences between the spleen colonization of mice infected with the wild-type *B. abortus* strain 2308 compared to the Δ*bsr6* strain, indicating that Bsr6 is dispensable for *B. abortus* host colonization in this model of infection. Nonetheless, our data clearly demonstrate that RpoE1 is required for the production of Bsr6 in *B. abortus*.

Regarding predictions of other sRNA levels linked to RpoE1, our initial testing for a regulatory relationship found no difference between the transcript levels of sRNAs in 2308 and the Δ*rpoE1* strain grown in a nutrient-replete, unstressed environment. However, the transcriptomic data utilized for the predictions were generated using *Brucella* strains cultured in the presence of oxidative stress (i.e., H_2_O_2_). As such, we analyzed RNA isolated from bacteria stressed with the addition of 5 mM H_2_O_2_, and we observed a modest ~20% increase in Bsr8 levels in the Δ*rpoE1* strain compared to wild-type 2308 (Fig. S1A and B). This is directionally in line with our prediction, though of a more modest effect than observed in the transcriptomic study. Given that the functional role of Bsr8 in *Brucella* had not been evaluated previously, we examined the contribution of Bsr8 to *Brucella* virulence using a mouse model of infection. A *B. abortus* D*bsr8* strain was constructed and used to infect BALB/c mice intraperitoneally, and spleen colonization was measured and compared to levels of mice infected with 2308 at 1-, 4-, and 8-weeks post-infection (Fig. S1C). From these experiments, we determined that Bsr8 is not required for the full virulence of *B. abortus* in the BALB/c model of chronic infection, and RpoE1 plays a limited role in the oxidative stress-responsiveness of Bsr8 production.

**Fig 4:**
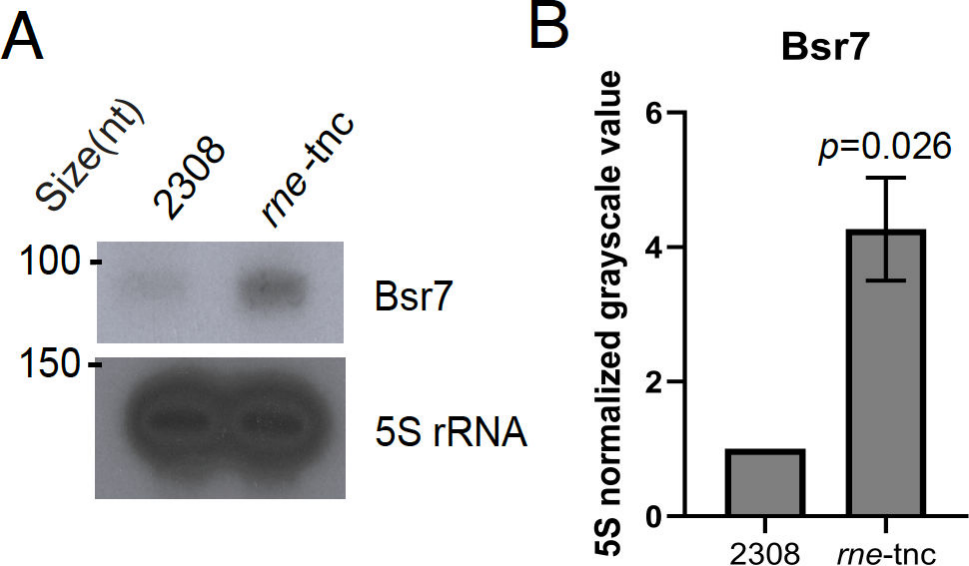
RNase E mediates Bsr7 turnover. Northern blot analyses and respective densitometry of 5S rRNA normalized mean grayscale value are displayed. Panel (**A**) shows a representative northern blot analysis of truncated Bsr7 levels in wild-type *B. abortus* 2308 and a strain harboring an RNase E truncation at the native locus (*rne*-tnc), and panel (**B**) depicts the densitometry of 5S rRNA normalized mean grayscale values from northern blot analyses carried out from at least three independent RNA isolations. Statistical analysis was carried out using a two-tailed *t*-test with two-sample equal variance, and the *P* value for each analysis is denoted in the bar graph.

**Fig 5:**
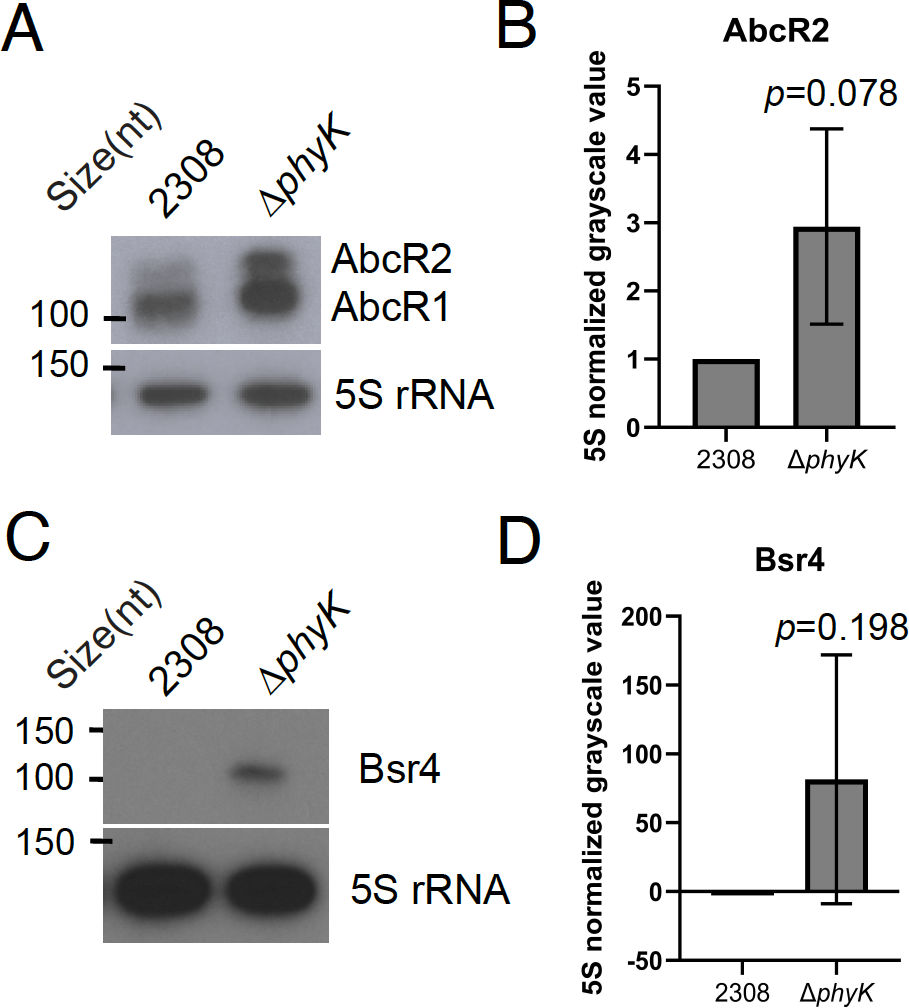
PhyK represses levels of AbcR2 and Bsr4. Northern blot analyses and respective densitometry of 5S rRNA normalized mean grayscale value are displayed. Panel (**A**) shows northern blot analysis of AbcR2 levels in wild-type *B. abortus* 2308 and a Δ*phyK* strain, and panel (**B**) depicts the densitometry of 5S rRNA normalized mean grayscale values from northern blot analyses carried out from at least three independent RNA isolations. Similarly, panel (**C**) shows northern blot analysis of Bsr4 levels in wild-type *B. abortus* 2308 and a Δ*phyK* strain, and panel (**D**) depicts the densitometry of 5S rRNA normalized mean grayscale values from northern blot analyses carried out from at least three independent RNA isolations. Statistical analysis was carried out using a two-tailed *t*-test with two-sample equal variance, and the *P* value for each analysis is denoted in the bar graph.

We next tested the prediction that RNase E is involved in mediating Bsr7 turnover, and indeed, northern blot analysis revealed substantially increased levels of Bsr7 in the *rne*-tnc strain compared to the wild-type strain (Fig. 4), which was in line with our predictions (Table 1). Finally, we tested the predictions for the histidine kinase PhyK. Our initial prediction indicated that AbcR2 levels would be significantly decreased in the Δ*phyK* genetic background (Table 1), but deletion of *phyK* had the opposite outcome, with AbcR2 levels being elevated in the Δ*phyK* mutant strain (Fig. 5A); however, these increases are not statistically significant, owing to significant variation between the biological replicates. The initial prediction also indicated elevated levels of Bsr4 in the Δ*phyK* strain compared to the wild-type strain 2308, and our northern blot analyses support this prediction (Fig. 5B). Nevertheless, substantial variation across independent replicates resulted in these differences not being statistically significant.

## DISCUSSION

Approximately 40 sRNAs have been identified within *Brucella* and validated by northern blot analysis. Of these, only a small number have been precisely mapped within the genome through identification of the 5′ and 3′ ends of the sRNA (11). The labor-intensive process of mapping sRNAs and the general lack of sRNAs annotated within reference genomes have heretofore precluded their examination in transcriptomic studies of *Brucella*, obfuscating the potential role of sRNAs in the observed experimental phenotypes.

To overcome this limitation, we sought a new method to identify potential regulatory relationships governing sRNA transcript levels. We generated an sRNA reference “genome” consisting of the general location of validated sRNAs concatenated together. Mapping the publicly available transcriptomic data to this reference allowed prediction of transcription factors controlling sRNA transcripts (Table 1). Since the exact transcript sequences are not known for most *Brucella* sRNAs, we opted for manual visual assessment of the mapped transcripts rather than automated read counting. To normalize for total reads mapped, we used overlaid bigwig files to provide an approximate fold change between the examined conditions over the length of the sRNA. We reanalyzed the transcriptomics from 14 studies and predicted 78 potential regulatory relationships for sRNAs (Table 1). Northern blot analysis over multiple types of transcription factors found agreement with four of five predicted regulatory relationships that we evaluated.

Northern blot analyses were in agreement with four of the predicted relationships: VjbR activating MavR, RpoE1 controlling Bsr6, RNase E mediating Bsr7 turnover, and PhyK repressing Bsr4 levels. One prediction, that the histidine kinase PhyK serves an activating role for AbcR2, was not confirmed. In fact, we found that PhyK had an opposite effect on AbcR2, as levels of the sRNA were substantially increased in the *B. abortus* Δ*phyK* strain compared to the wild-type strain 2308, indicating PhyK plays a repressive role in modulating levels of AbcR2 (Fig. 5). Collectively, given these findings along with those of Fig. 1, this method will likely be effective in assessing sRNA transcript changes in *Brucella* species, but also in other bacterial species, and further refinement may be beneficial. Specifically, the results showing RpoE1-linked control of MavR (Fig. 2D), along with the comparison to the Saadeh et al. analysis (Fig. 1), indicate that potentially biologically meaningful relationships exist below our conservatively selected cut-off of fourfold change for analysis. Further work to empirically relate northern blot fold-change levels and transcriptome levels may aid in selecting a fold-change cutoff that is more sensitive and specific. As more sRNAs are directly mapped, it will also be possible to better quantify the transcript levels of the sRNAs using read counting.

It is unclear exactly why the AbcR2 of the predictions for PhyK made from the *Brucella ovis* transcriptome did not translate to *B. abortus*, but there are some possible explanations for these discrepancies. Most obviously, we cannot rule out that either PhyK or the sRNA may exhibit different regulation in the species-specific backgrounds, especially given that PhyK is an orphan histidine kinase with an uncharacterized regulatory pathway (20). The relative variability of sRNA transcript levels across *Brucella* species has only been minimally documented, but our lab has found that the transcript for MavR is differentially expressed between *B. abortus*, *Brucella melitensis*, and *Brucella suis* grown in identical conditions (30). Additionally, the original transcriptome set was collected from cultures grown on solid agar, while our samples came from late exponential phase liquid cultures, and as such, growth or medium differences may explain the inconsistency. Finally, it is possible that the prediction itself was simply a false positive from that specific transcriptome study and would not necessarily be replicated even in *B. ovis*. More extensive probing of cross-species predictions and more systemic analysis of sRNA transcript levels may aid in determining the limitations of our proposed prediction methods.

With regard to specific regulatory relationships we found, the sRNA MavR is essential for full virulence of *B. abortus* in a mouse model and acts to suppress translation of peptidoglycan synthesis protein MurF (30). Similarly, the transcriptional regulator VjbR is involved in the quorum-sensing system of *Brucella*, and deletion of *vjbR* results in attenuation in multiple models of infection (31–33). The regulation of MavR transcript level by VjbR is striking (Fig. 2), and it is possible that the lowered MavR levels contribute to the virulence defect observed in Δ*vjbR* strains. The mechanism of the regulation of *mavR* by VjbR remains unexplored in this analysis, but a known VjbR binding site occurs ~250 bp downstream of *mavR* (21). This general area is also bound by the H-NS-like protein MucR (34). Given the lack of the VjbR binding site in the promoter area of *mavR*, we suggest that VjbR acts to counter the MucR silencing, allowing expression of *mavR* and subsequent control of MurF. Though we did not directly test this relationship here, we would predict based on the function of MavR that MurF levels would be higher in the Δ*vjbR* mutant.

RpoE1 is a sigma factor acting as part of the general stress response of *Brucella*, where it is activated downstream of PhyR in response to general stress sensed by the LovhK system (29). RpoE1 is critical for the maintenance of chronic infection in *Brucella* and directly or indirectly modulates the expression of several virulence factors (27–29). We show that the sRNA Bsr6 requires RpoE1 for its expression (Fig. 3) and, tellingly, there is a clear RpoE1 consensus binding box within the promoter of Bsr6 (Fig. 3B). The role of Bsr6 in *Brucella* physiology is unknown, though given its association with RpoE1, we were curious if it is required for full virulence in the mouse model. Deletion of *bsr6* still resulted in full splenic virulence, at least at the time points we examined (Fig. 3C). Our transcriptomic analyses also suggested that the transcriptional regulator BaaR may also control Bsr6 levels (Table 1). BaaR is an IclR-family regulator indirectly activated by RpoE1 and is involved with adipic acid transport and metabolism (15). A known BaaR binding box is not present in the *bsr6* promoter, so the exact regulatory mechanism between BaaR and Bsr6 remains to be discovered, as well as the broader relationship between RpoE1, BaaR, and Bsr6 in *Brucella* physiology.

We found that RpoE1 also acts to minimally repress Bsr8 in the presence of H_2_O_2_ stress (Fig. S1). The role of Bsr8 in *Brucella* physiology is also unknown, but our data utilizing a mouse model of infection indicates that Bsr8 is non-essential for full splenic virulence. Additional characterization to reveal the role of Bsr8 in *Brucella* biology and how Bsr8 fits into the RpoE1 regulon is needed.

PhyK is a histidine kinase in the same genomic region as the general stress response of *Brucella,* but its direct role in this pathway is unclear. Nevertheless, it is a major regulator of membrane transport and energetics that promotes cell envelope integrity (20). Deletion of *phyK* in *B. ovis* resulted in dramatic transcriptional change, and we also observed multiple sRNAs with predicted dysregulation. The method of PhyK regulation of sRNAs remains to be identified. The final type of regulatory mechanism we tested was transcript control by RNases, which degrade the sRNA transcript, typically when it is bound to its conjugate mRNA target (22). We found that Bsr7 is controlled by RNase E, and the exact role of Bsr7, as well as the relationship between RNase E and Bsr7, in *Brucella* biology, remains to be elucidated.

In summary, we developed a method for discovery of transcription factors controlling sRNA expression by creating a custom reference of known sRNAs and mapping transcriptomic data to it. Northern blot analyses for a subset of these predictions across a range of regulatory mechanisms showed broad agreement with the predictions. As such, this method may prove useful to the wider bacteriology community for extracting more data from transcriptomic approaches and tracking sRNA expression.

## MATERIALS AND METHODS

### Bacterial growth and strains

Indicated *Brucella* strains were grown on Schaedler blood agar with 5% bovine blood (Quad Five) and inoculated into brucella broth to grow at 37°C, shaking at 200 rpm unless otherwise indicated. *Escherichia coli* strains were grown on tryptic soy agar and inoculated into lysogeny broth to grow at 37°C, shaking at 200 rpm. Where appropriate, kanamycin 45 ug/mL was supplemented into the media. A full list of sources of strains is available in the supplemental files.

### Bioinformatic approach

We generated a custom FASTA file containing known *Brucella* sRNAs by concatenating the approximate genomic location of known sRNAs together. Because many of the sRNAs are not mapped, and the length of probes for northern blot analysis varied considerably, the amount of genomic area selected for concatenation of each sRNA was determined manually. Most often, the portion selected was the inter-intergenic region; however, for some sRNAs that appeared to overlap with flanking protein-encoding genes or were detected as trans-encoded, a portion of the flanking gene was selected in proportion to the expected size of the sRNA. To allow easier customization for future studies, each selected portion of the genome selected for concatenation was separated by an inserted block of 25 “G” bases between regions within the mapping file. Separate files were made for *Brucella abortus* 2308, *Brucella melitensis* 16M, *Brucella suis* 1330, and *Brucella ovis* ATCC25840. These files are available in the supplement. Publicly available sequence read archive files of RNA-seq experiments were downloaded from the National Center for Biotechnology Information (NCBI) or the European Nucleotide Archive (ENA) and mapped to the generated reference files via bowtie2 (35). Where required, BAM files uploaded to NCBI or ENA were first converted to fastq via DeepTools (36). Files were mapped to their respective species, with the single *Brucella canis* transcriptome mapped to the *B. suis* file. Following mapping, reads were sorted and indexed with Samtools (37), and the relative read-normalized expression was calculated through the generation of bigwig files with DeepTools using the default bin size. The mapped reads and bigwig files were then visualized with Integrated Genome Viewer (38) and qualitatively examined. sRNAs showing an approximately fourfold change between a control and experimental condition were recorded as predictions.

### Northern blot analysis

Northern blot analysis was conducted as previously described (14). Briefly, *Brucella* strains were grown to an optical density of approximately 1 at 600 nM wavelength. The bacterial cultures were then killed and stabilized with equal volumes of ethanol and acetone, and nucleic acids were isolated via Trizol extraction. The sRNA transcript was visualized by probing with a short complementary oligonucleotide radiolabeled with ^32^P and followed by autoradiography. The specific probes used are available in the supplementary files.

Densitometry was performed using Fiji (39). Eight-bit images were generated from film scans, and the entire image was qualitatively adjusted to enhance contrast. The largest observed band was used to generate a selection area, and the mean gray area was measured for each band using the standard selection area, along with a control area with no bands. Inverted pixel density within the selection area was calculated by subtracting the mean value from the maximum measurable value. These net measurements were then calculated by subtracting the control area from the inverted pixel density. sRNA bands were normalized to their respective 5S rRNA loading control bands. These values were then normalized to those of the wild-type measurement to calculate the fold change.

### Construction of deletion mutants

In-frame, markerless deletions of *bsr6* and *bsr8* were generated via allelic exchange using pNTSP138 as the exchange vector. An approximately 1 kb region upstream and downstream of *bsr6* or *bsr8* was amplified from *B. abortus* 2308 genomic DNA using polymerase chain reaction. Primers utilized are in the supplementary files. The ends of the two regions were digested with compatible restriction enzymes (BamHI and PstI) and ligated into similarly digested pNTSP138. The resulting Δ*bsr* construct plasmids were confirmed via Sanger sequencing and electroporated into *B. abortus* 2308. The Δ*bsr* strains were selected for on kanamycin plates, and merodiploids were resolved on 10% sucrose agar plates. Deletion of *bsr6* or *bsr8* was confirmed via polymerase chain reaction (PCR) on the resulting *B. abortus* strain, and northern blot analysis confirmed the absence of the Bsr transcript. Deletion of *rpoE1* and *phyK* was achieved via electroporation of the deletion constructs (kind gifts from Dr. Sean Crosson) into *B. abortus* 2308, followed by similar confirmation via PCR.

### Mouse infection assay

Complete methods for mouse infection in line with ARRIVE 2.0 guidelines are available in the supplemental materials. In brief, five female BALB/c mice per strain per time point were intraperitoneally infected with approximately 100,000 colony-forming units (CFU) of the indicated *B. abortus* strain and housed in an appropriate ABSL3 environment. At the time points indicated, mice were humanely euthanized, and individual spleens were aseptically removed, homogenized, and the homogenates were plated for enumeration of CFUs.

### Biosecurity and material availability 

All work with live *Brucella abortus* was conducted in a BSL3 environment, in line with federal regulations in place at the time of experiments. The physical materials generated are available upon request.

## SUPPLEMENTAL MATERIAL

Supplemental data spectrum.01909-25-s0001.xlsx Transcriptome alignment to sRNA maps.

Supplemental material spectrum.01909-25-s0002.pdf Fig. S1; Supplemental text.

ASM does not own the copyrights to Supplemental Material that may be linked to, or accessed through, an article. The authors have granted ASM a non-exclusive, world-wide license to publish the Supplemental Material files. Please contact the corresponding author directly for reuse.
