# Supplementary material for: A method for discovery of transcription factors controlling Brucella sRNAs
Source: Microbiol Spectr. 2025 Nov 13;14(1):e01909-25. doi: 10.1128/spectrum.01909-25 (PMC12772402; doi:10.1128/spectrum.01909-25)
Supplement: Supplemental material — Fig. S1; Supplemental text. [file spectrum.01909-25-s0002.pdf]

A method for discovery of transcription factors controlling *Brucella* sRNAs

SUPPLEMENTAL MATERIAL

Mitchell T. Caudill[1], Jillian R. Marshall[1], Clayton C. Caswell[1,*]

[1]Center for One Health Research, Department of Biomedical Sciences and Pathobiology, VA-MD College of Veterinary Medicine, Virginia Tech, Blacksburg, VA, United States.

In Depth Methods of Mouse Studies

<u>Experiments Conducted for Figure 3C</u>

This experiment was conducted to determine the relative virulence of bacterial strains (Δ*bsr6*) compared to a wildtype control infection (2308) at 1-, 4-, and 8-weeks after infection. The experimental unit was individual mice. Five approximately 6-week-old female BALB/c mice per strain per time point (30 mice total, 5 per group per time point) were used. Sample sizes were selected based on modified use of mouse models within the field. No *a priori* criteria were set for including or excluding animals for the experiment, and no data from mice were excluded from the analysis.

Mice were allowed to acclimatize in ABSL-1 housing after arrival before being moved into BSL-3 conditions for infection and housing. Mice were injected intraperitoneally with ~100,000 bacterial colony forming units (CFUs) of *Brucella* suspended in sterile PBS in order of convenience with experimentalists unblinded to injection conditions and remaining unblinded throughout the experiment. Mice were then cohoused by strain within a single housing unit in an ABSL3 environment, with cages placed via convenience. During the experiment, mice were provided *ad libitum* food and water, sufficient nesting material, and enrichment. No adverse effects were observed, and no humane endpoints (veterinarian recommendation following observation of lethargy, anorexia, labored breathing or ruffled coat) were exercised.

At time points indicated in the analysis, mice were humanely euthanized in order of convenience with carbon dioxide followed by cervical dislocation in accordance with IACUC approved protocols. The outside of the mice was sterilized with 70% alcohol, and the spleens were aseptically removed via sharp dissection. Spleens were homogenized and serial dilutions were plated on agar plates. Colony forming units were then counted approximately three days after plating, with the number of bacterial colonies serving as the experimental outcome.

Statistical analysis was conducted using GraphPad Prism (v9.5.1). The bacterial colony counts were statistically tested via a repeated measures two-way ANOVA with post-hoc Tukey's multiple comparisons test to determine differences between groups. Additional tests of normality were not conducted. Summary statistics of the data presented in Figure 3 are reproduced below:

1 **Week**

Strain	Mean ($\log_{10}$ cfu)	Standard Deviation
2308	5.69	0.23
Δ*bsr6*	5.60	0.30

4 Weeks

Strain	Mean ($\log_{10}$ cfu)	Standard Deviation
2308	6.70	0.26
Δ*bsr6*	6.67	0.20

8 Weeks

Strain	Mean ($\log_{10}$ cfu)	Standard Deviation
2308	6.87	0.56
Δ*bsr6*	6.85	0.33

Experiments conducted for Figure 4C

This experiment was conducted to determine the relative virulence of bacterial strains (Δ*bsr6*) compared to a wildtype control infection (2308) at 1, 4, and 8 weeks after infection. Procedures were the same as Figure 3 above. Summary statistics of the data presented in Figure 4 are reproduced below:

1 Week

Strain	Mean ($\log_{10}$ CFU)	Standard Deviation
2308	6.72	0.24
Δ*bsr8*	6.86	0.22

4 Weeks

Strain	Mean ($\log_{10}$ CFU)	Standard Deviation
2308	6.59	0.33
Δ*bsr8*	6.97	0.24

8 Weeks

Strain	Mean ($\log_{10}$ CFU)	Standard Deviation
2308	6.03	0.35
Δ*bsr8*	5.80	1.08

**Strains**	**Genotype Description**	**Source**
Brucella abortus		
2308	Wildtype strain	Gift of R. Martin
Roop II		
Δ*bsr6*	In frame, markerless deletion of sRNA bsr6	This project
Δ*bsr8*	In frame, markerless deletion of sRNA bsr8	This project
Δ*hfq*	In frame, markerless deletion of RNA chaperone Hfq	Caswell, 2012
Δ*phyK*	In frame, markerless deletion of transcriptional regulator phyK	This project

Δ*rpoE1*	In frame, markerless deletion of sigma factor RpoE1	Kim, 2014
rne-tnc	In frame truncation of RNase E	Sheehan, 2020
Δ*vjbR*	In frame, markerless deletion of transcriptional regulator VjbR	Caudill, 2025

E. coli

DH5a	For maintainance and propagation of plasmids	

Plasmids

pNPTS138		M. R. K. Alley, unpublished
pΔ*bsr6*	pNPTS138::1kb sequences flanking bsr6	This project
pΔ*bsr8*	pNPTS138::1kb sequences flanking bsr6	This project
pΔ*rpoE1*	pNPTS138::1kb sequences flanking RpoE1	Gift of Sean Crosson
pΔ*phyK*	pNPTS138::500bp sequences flanking BAB1_1669	Gift of Sean Crosson

Primers

**Name**	**Sequence**	**Restriction Site (In bold)**
bsr6-Up-For	GC**GGATCC**TGGAAACGCTGCTGCCTGA	BamHI
bsr6-Up-Rev	GGGTCACGGTCGTCATATCC	
bsr6-Down-For	TTTTTCGGGAATATCGCCCAGG	
bsr6-Down-Rev	GC**CTGCAG**GGAAGCAGAACCAGACGCAGCA	Pst1
bsr6-con-For	GCAGGAAGAGATCGCGCGTT	
bsr6-con-Rev	TGTTTTCGAGCACGATCCCC	
bsr8-Up-For	AG**GAATTC**CGCGTTGACGACGCCCTGAA	EcoRI
bsr8-Up-Rev	AGTCCATCTCGTCGTCTCAACCG	

bsr8-Dn-For	CTTTCAGGCTTCTCATGCACTCC	
bsr8-Dn-Rev	AG**GCTAGC**CGTGGCGATGTGGATCGC	NheI
M13 F	GTTTTCCCAGTCACGAC	
M13 R	CAGGAAACAGCTATGAC	
PhyK Con F	GCGTTCGCTCGGCTTCGAGATCGTT	
PhyK Con R	GCGCTTCTGGTTATTTCGCTCATCG	
**Northern Probes**		
AbcR1/2	GGCAAACCTCCAGAGGGGAACACT	
MavR	GGAGGGTGCTTCCACGGAGAAGATTGTCAAAA	
Bsr4	TCCATCCTCCCAAGACTTCACCGCGTGA	
Bsr6	CCGTTGGACAGTTAATGCGGCATTG	
Bsr7	GTCAAACAGAGTGGCTAAACCACTCGGTAA	
Bsr8	CCGAGACAGGACTTCCAACGTGC	

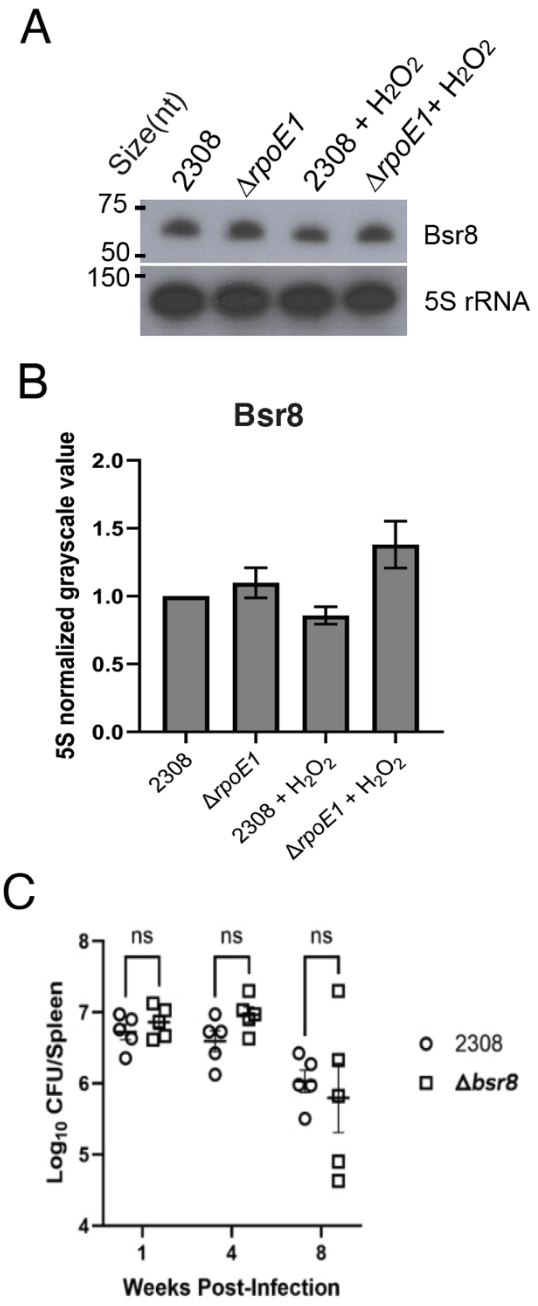

Figure S1. Bsr8 is not required for the full virulence of *Brucella abortus*.

Northern blot analyses and their respective densitometry of 5S rRNA normalized mean greyscale value are displayed. Panels A&B shows a representative northern blot analysis of Δ*rpoE1* for Bsr8 transcript levels with and without H_2O_2 and the associated densitometry from two biological replicates. Panel C demonstrations splenic colony forming units at the indicated

time points for five female BALB/c mice per strain infected intraperitoneally. Statistical testing consisted of a two-way ANOVA with post-hoc Tukey’s multiple comparison. NS indicates no statistical significance.

Supplemental References

1. Budnick JA, Sheehan LM, Benton AH, Pitzer JE, Kang L, Michalak P, Roop RM 2nd, Caswell CC. Characterizing the transport and utilization of the neurotransmitter GABA in the bacterial pathogen Brucella abortus. PLoS One. 2020 Aug 26;15(8):e0237371. doi: 10.1371/journal.pone.0237371. PMID: 32845904
2. Caswell CC, Gaines JM, Roop RM 2nd. The RNA chaperone Hfq independently coordinates expression of the VirB type IV secretion system and the LuxR-type regulator BabR in Brucella abortus 2308. J Bacteriol. 2012 Jan;194(1):3-14. doi: 10.1128/JB.05623-11. Epub 2011 Oct 21. PMID: 22020650
3. Caudill MT, Stoyanof ST, Caswell CC. Quorum sensing LuxR proteins VjbR and BabR jointly regulate *Brucella abortus* survival during infection. J Bacteriol. 2025 Mar 20;207(3):e0052724. doi: 10.1128/jb.00527-24. Epub 2025 Feb 27. PMID: 40013834
4. Chen, X. Cell envelope regulatory two-component systems in *Brucella ovis*. PhD thesis completed at Michigan State University under the supervision of Crosson S, Abramovitch R, Shames S, Hergholz T. 2024. doi:10.25335/k277-v223.
5. Chen X, Alakavuklar MA, Fiebig A, Crosson S. Cross-regulation in a three-component cell envelope stress signaling system of *Brucella*. mBio. 2023 Dec 19;14(6):e0238723. doi: 10.1128/mbio.02387-23. Epub 2023 Nov 30. PMID: 38032291
6. de la Garza-García JA, Ouahrani-Bettache S, Lyonnais S, Ornelas-Eusebio E, Freddi L, Al Dahouk S, Occhialini A, Köhler S. Comparative Genome-Wide Transcriptome Analysis of Brucella suis and Brucella microti Under Acid Stress at pH 4.5: Cold Shock Protein CspA and Dps Are Associated With Acid Resistance of B. microti. Front Microbiol. 2021 Dec 13;12:794535. doi: 10.3389/fmicb.2021.794535. PMID: 34966374

7. Herrou J, Czyż DM, Fiebig A, Willett JW, Kim Y, Wu R, Babnigg G, Crosson S. Molecular control of gene expression by *Brucella* BaaR, an IclR-type transcriptional repressor. J Biol Chem. 2018 May 11;293(19):7437-7456. doi: 10.1074/jbc.RA118.002045. Epub 2018 Mar 22. PMID: 29567835
8. Hop HT, Arayan LT, Reyes AWB, Huy TXN, Min W, Lee HJ, Son JS, Kim S. Simultaneous RNA-seq based transcriptional profiling of intracellular Brucella abortus and B. abortus-infected murine macrophages. Microb Pathog. 2017 Dec;113:57-67. doi: 10.1016/j.micpath.2017.10.029. Epub 2017 Oct 18. PMID: 29054743.
9. Kleinman CL, Sycz G, Bonomi HR, Rodríguez RM, Zorreguieta A, Sieira R. ChIP-seq analysis of the LuxR-type regulator VjbR reveals novel insights into the Brucella virulence gene expression network. Nucleic Acids Res. 2017 Jun 2;45(10):5757-5769. doi: 10.1093/nar/gkx165. PMID: 28334833
10. Kim HS, Willett JW, Jain-Gupta N, Fiebig A, Crosson S. The Brucella abortus virulence regulator, LovhK, is a sensor kinase in the general stress response signalling pathway. Mol Microbiol. 2014 Nov;94(4):913-25. doi: 10.1111/mmi.12809. Epub 2014 Oct 19. PMID: 25257300
11. Liu W, Dong H, Li J, Ou Q, Lv Y, Wang X, Xiang Z, He Y, Wu Q. RNA-seq reveals the critical role of OtpR in regulating Brucella melitensis metabolism and virulence under acidic stress. Sci Rep. 2015 Aug 5;5:10864. doi: 10.1038/srep10864. PMID: 26242322
12. Rodríguez MC, Viadas C, Seoane A, Sangari FJ, López-Goñi I, García-Lobo JM. Evaluation of the effects of erythritol on gene expression in Brucella abortus. PLoS One. 2012;7(12):e50876. doi: 10.1371/journal.pone.0050876. Epub 2012 Dec 14. PMID: 23272076

13. Sheehan LM, Budnick JA, Fyffe-Blair J, King KA, Settlage RE, Caswell CC. The Endoribonuclease RNase E Coordinates Expression of mRNAs and Small Regulatory RNAs and Is Critical for the Virulence of Brucella abortus. J Bacteriol. 2020 Sep 23;202(20):e00240-20. doi: 10.1128/JB.00240-20. PMID: 32747427
14. Sun J, Dong H, Peng X, Liu Y, Jiang H, Feng Y, Li Q, Zhu L, Qin Y, Ding J. Deletion of the Transcriptional Regulator MucR in *Brucella canis* Affects Stress Responses and Bacterial Virulence. Front Vet Sci. 2021 Jun 25;8:650942. doi: 10.3389/fvets.2021.650942. PMID: 34250056
15. Sun D, Liu Y, Peng X, Dong H, Jiang H, Fan X, Feng Y, Sun J, Han K, Gao Q, Niu J, Ding J. ClpP protease modulates bacterial growth, stress response, and bacterial virulence in Brucella abortus. Vet Res. 2023 Aug 23;54(1):68. doi: 10.1186/s13567-023-01200-x. PMID: 37612737
16. Varesio LM, Willett JW, Fiebig A, Crosson S. A Carbonic Anhydrase Pseudogene Sensitizes Select *Brucella* Lineages to Low CO_2 Tension. J Bacteriol. 2019 Oct 21;201(22):e00509-19. doi: 10.1128/JB.00509-19. PMID: 31481543
17. Zhang H, Sun T, Cao X, Wang Y, Ma Z, Wang Y, Yang N, Xu M, Deng X, Li H, Wang B, Yi J, Wang Z, Zhang Q, Chen C. Scanning iron response regulator binding sites using Dap-seq in the Brucella genome. PLoS Negl Trop Dis. 2023 Jul 17;17(7):e0011481. doi: 10.1371/journal.pntd.0011481. PMID: 37459300
